# Evaluation of Correlation of Cell Cycle Proteins and Ki-67 Interaction in Paranasal Sinus Inverted Papilloma Prognosis and Squamous Cell Carcinoma Transformation

**DOI:** 10.1155/2014/634945

**Published:** 2014-06-12

**Authors:** Yung-An Tsou, Hung-Jin Huang, Tang-Chuan Wang, Chih-Jaan Tai, Chuan-Mu Chen, Calvin Yu-Chian Chen

**Affiliations:** ^1^Department of Otolaryngology Head and Neck Surgery, China Medical University, Taichung 40402, Taiwan; ^2^Department of Medicine, School of Medicine, China Medical University, Taichung 40402, Taiwan; ^3^Department of Life Sciences, Agricultural Biotechnology Center, National Chung Hsing University, Taichung 402, Taiwan; ^4^Department of Chinese Pharmaceutical Sciences and Chinese Medicine Resources, College of Pharmacy, China Medical University, Taichung 40402, Taiwan; ^5^Department of Biomedical Informatics, Asia University, Taichung 41354, Taiwan; ^6^Computational and Systems Biology, Massachusetts Institute of Technology, Cambridge, MA 02139, USA; ^7^Research Center for Chinese Medicine and Acupuncture, China Medical University, Taichung 40402, Taiwan

## Abstract

The recurrent sinonasal inverted papilloma (IP) could be transformed to sinonasal squamous cell carcinoma. We use protein expression patterns by immunohistochemical method to see whether the expression of p53, p16, p21, and p27 belongs to cell-cycle-regulators and PCNA (proliferating cell nuclear antigen) and Ki-67 the proliferation markers in sixty patients with sinonasal inverted papilloma, and 10 of them with squamous cell carcinoma transformation. Significantly elevated levels of Ki-67, p27, and PCNA in IP with squamous cell carcinoma transformation of sinonasal tract compared with inverted papilloma were revealed. No variation of p16, p21, PLUNC (palate, lung, and nasal epithelium clone protein) and p53 expression was correlated to sinonasal IP malignant transformation by multivariate survey. However, we found elevated PLUNC expression in IPs with multiple recurrences. Finally, we found that PCNA, p27 may interact with CDK1 which promote IP cell proliferation and correlate to sinonasal squamous cell carcinoma. Ki-67 could work throughout the cell cycles to cause malignant transformation. In conclusion, this is a first study showing the correlation of Ki-67, PCNA interacted with CDK1 might lead to malignant transformation. Elevated PLUNC expression in the sinonasal IPs was related to multiple recurrences in human.

## 1. Introduction

The inverted papilloma (IP) is a type of tumor in which surface epithelial cells grow downward into the underlying supportive tissue. The bladder, renal pelvis, ureter, urethra, nose, and paranasal sinuses are all possible areas for IP occurrence [1]. Epistaxis and nasal obstruction with facial pain or headache attacked when the nose or sinuses mucosa bear the IP [2]. Although the IP originating from the outlining respiratory membrane belongs to a benign epithelial neoplasm, the local invasiveness, higher recurrence rate, and malignant transformation make it difficult to treat [3]. The malignant transformation rate is 5–10% and many of them are synchronous presenting with squamous cell carcinoma [4]. PCNA (proliferating cell nuclear antigen) acts as an antigen expression in the cell nuclei in the phase of DNA synthesis during cell cycle and considers the cancer prognosis, but the relationship to IP is still controversial [5].

Cyclin and cyclin dependent kinase (CDK) partake the important roles in cell cycle during proliferation and affect different cell cycle phases [6–9]. The CDK1 is a very crucial initiator for cell proliferation and malignant transformation factor [10]. On the contrary, the p21, P27, and CDK inhibitors, limited CDKs and arrest the cell cycle [8]. The p21 is an inhibitor of G1 cell phase CDKs which restrain cells entry into S phase. The p27 could also bind to CDKs and act as CDKI as p21. The p27 interacts with cyclin E-CDK2, cyclin A-CDK2, and cyclin D1-CDK4 complexes, affecting cell proliferation and apoptosis [6, 7].

The proliferation marker Ki-67 antigen, detected with monoclonal antibody MIB-1, is expressed in all G1, S, G2, and M cell phases except G0 [8]. Ki-67 expression also correlated to the tumor behavior, pathologic tumor grade, and early recurrence in various carcinomas [11–15].

Concerning the IPs transformed or synchronous with cancers, we also do the p16, p53, Ki-67, and PLUNC (palate, lung, and nasal epithelium clone protein) IHC study. The p16 is a tumor suppressor protein decelerating G1 to S cell phase and prevent of malignant transformation [8]. PLUNC is an innate immune material that has an anticancer effect for nasopharyngeal cancer but no studies revealed its relevance to sinonasal IPs [16].

PCNA, p53, p21, p27, and Ki-67 surveys were done to see whether they were correlating to tumor extents and the treatment outcome of sinonasal IPs [8].

The aim of this study is to investigate the roles of cell-cycle-regulators p53, p21, p27, proliferation marker Ki-67, p16, PCNA, and innate immune material PLUNC in recurrence and malignant transformation for sinonasal IPs. We had also surveyed whether possible predicted factors of Ki-67, PCNA, and p27 correlate to CDKs by computational simulation finally to give a possible explanation to the mechanism of Ki-67, PCNA, and p27 to the CDKs in IPs prognosis.

## 2. Materials and Methods

From Department of China Medical University Hospital from January 2000 to June 2010, 60 cases of sinonasal IPs and 10 cases of IPs with squamous cell carcinoma transformation were collected and reviewed from the medical records in a retrospective manner. All of the tissues fixed in 10% formalin and prepared in paraffin were used for IHC studies by avidin-biotin-peroxidase complex method. Mouse monoclonal antibodies (mAb), anti-p16 (Neomarkers, DCS-50, 200 mg/L), anti-p21 (Neomarkers, MS 387-P, 200 mg/L), anti-p27 (Neomarkers, MS 256-P, 200 mg/L), anti-p53 (Neomarkers, RM 9105-S), Ki-67 (Neomarkers RM 9106-S) PCNA, and PLUNC (Santa Cruz Biotechnology, Santa Cruz, CA, USA) were used for immunohistochemistry through the streptavidin-biotin peroxidase method [11]. Xylene was used for deparaffinizing all of the tissue sections and then rehydrated by alcohol series, and finally saturated in distilled water. Then the tissue was shifted into phosphate-buffered saline with adding 0.3% solution of hydrogen peroxidase to block the endogen peroxidase activity at room temperature for 10 min, then Tris buffer rinsed thereafter. The sections were then boiled by a microwave oven for 15 min in citrate buffer solution (10 mmol/L; pH, 6.0). Primary antibodies p53, p21, p27, p16, PCNA, Ki-67, and PLUNC was applied one by one for 60 min at room temperature [11].

Then we add linking antibody and streptavidin peroxidase complex (DAKO LSAB Kit, K-0675; Carpinteria, CA) for 15 min at room temperature. The 0.05% diaminobenzidine tetrahydrochloride (DAB) was used for 15 min for tissue stain and finally washed twice by the Tris buffer [8, 11].

The sections were counterstained with Mayer's hematoxylin after washing by deionized water. The immunostained nuclei were quantified in each case. All counting was performed under a standard light microscope in 1000x field to evaluate positive nuclei/total number of cells. Ten fields or at least 500 cells were counted on each section. Tumor sections were considered negative if staining was absent or present in <10% of tumor cells. A score of 1+ was given when 10–30% of the cells were positive to the reaction. A score of 2+ was given when 30–50% of the cells were positive to the reaction. A score of 3+ was given when** >**50% of the cells were positive to the reaction, respectively (Tables 1 and 2) [17]. Statistical significance was analyzed using the Pearson's chi-squared test or Fisher exact test for univariate analysis and multiple logistic regression test was used for multivariate analysis. Results were considered statistically significant when the *P* value was** <**0.05.

The protein-protein docking was carried out by ZDOCK program [18] for analyzing the three possible prognostic factors for malignant transformation bound to CDK1. To render the possible mechanism to what we found in this study, we calculate the interaction of the Ki-67, p27, and PCNA to CDK1 by computational biology. We further utilized GROMACS 4.5.5 program [19] to observe the stability of the complexes after ZDOCK binding predications. The environment of MD system was set in the TIP3P water modeling with 1.2 nm distance water box which contained Na and Cl ions in the concentration of 0.145 M NaCl for system neutralization. First, we set 5,000-cycle steps in the steepest descent algorithm for energy minimization. Secondly, the constant temperature dynamics (NVT type) conditions were employed to provide MD simulation for equilibration and performed in 1 ns time period. In the final step, the constant pressure and temperature dynamics (NPT type) were set for the production run in 5000 ps time period. The temperature of the system during the simulation process was set as 310 K. For trajectory analysis, we employed software GROMACS 4.5.5 to count the root mean square deviation (RMSD) and radius of gyration (Rg), respectively. Series of MD conformation data was surveyed every 20 ps of all production runs.

## 3. Results

**Figure 1:**
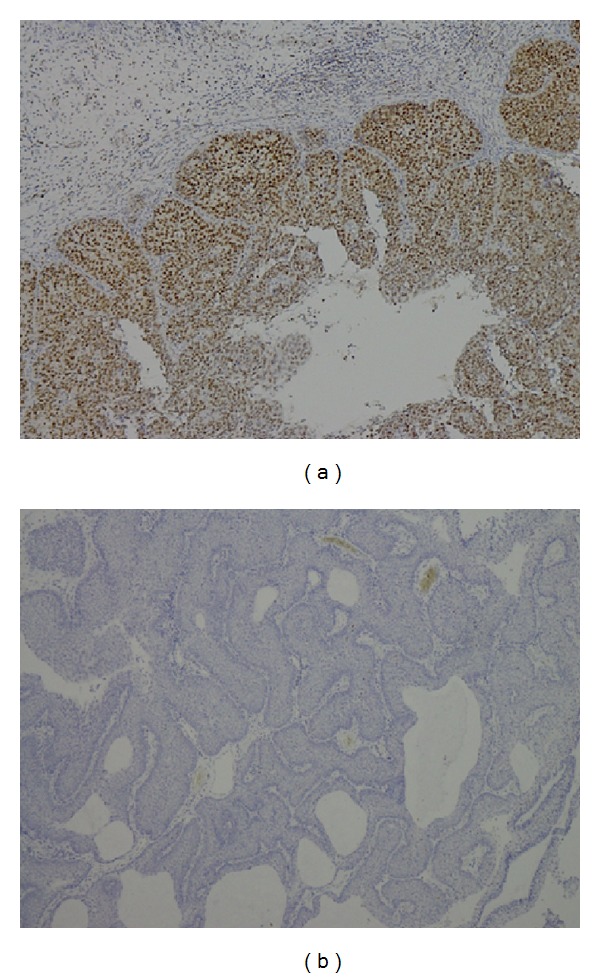
The PCNA IHC (a) expression +++ and (b) 0 in IPs.

**Figure 2:**
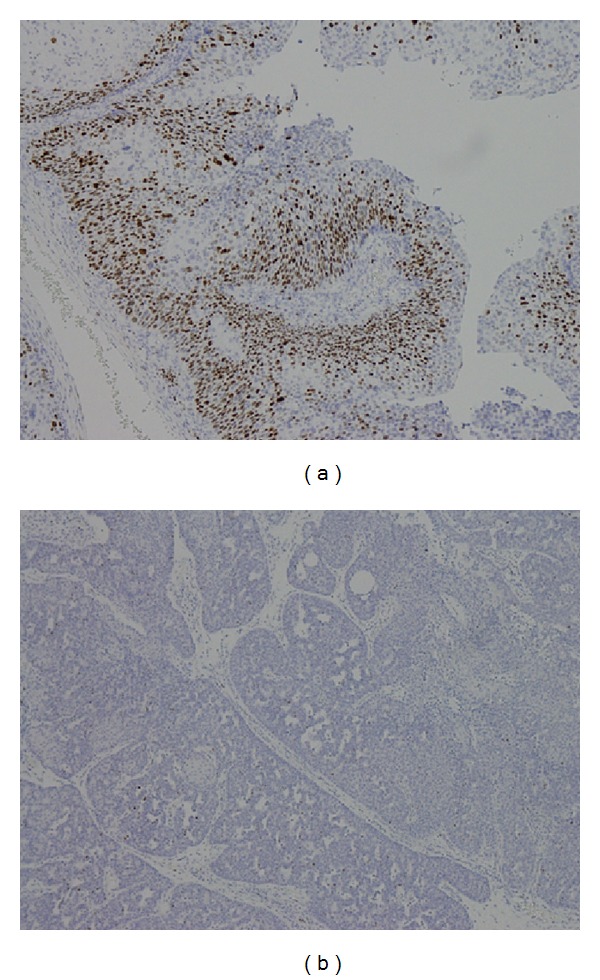
The Ki67 IHC (a) expression +++ and (b) 0 in IPs.

**Figure 3:**
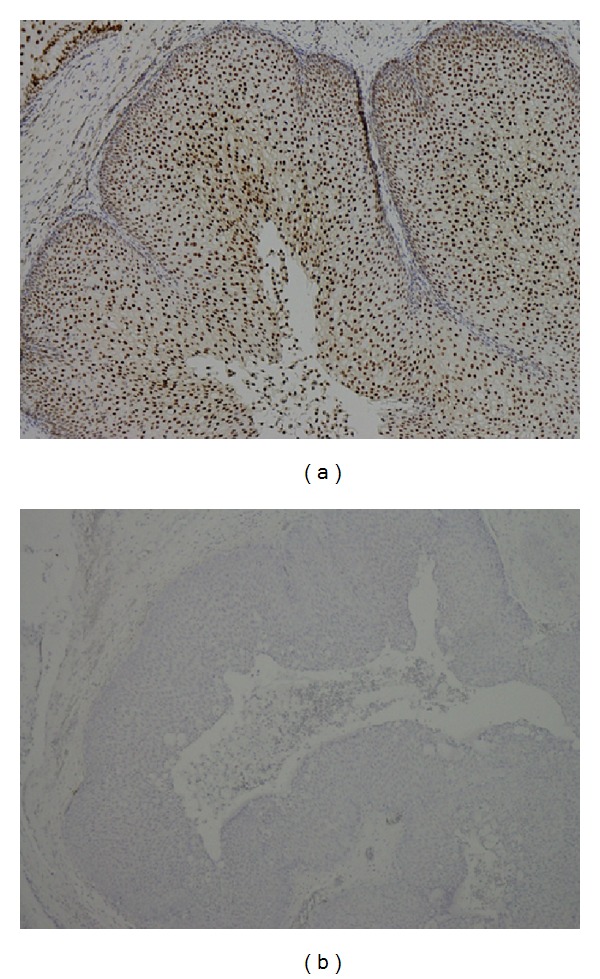
The p27 IHC (a) expression +++ and (b) 0 in IPs.

**Figure 4:**
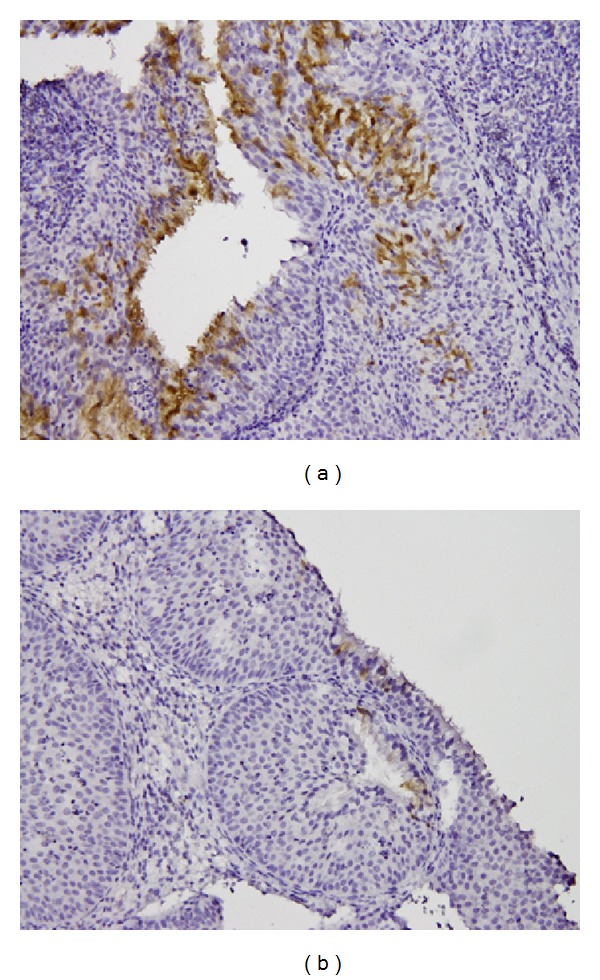
The PLUNC IHC (a) expression +++ and (b) 0 in IPs.

**Figure 5:**
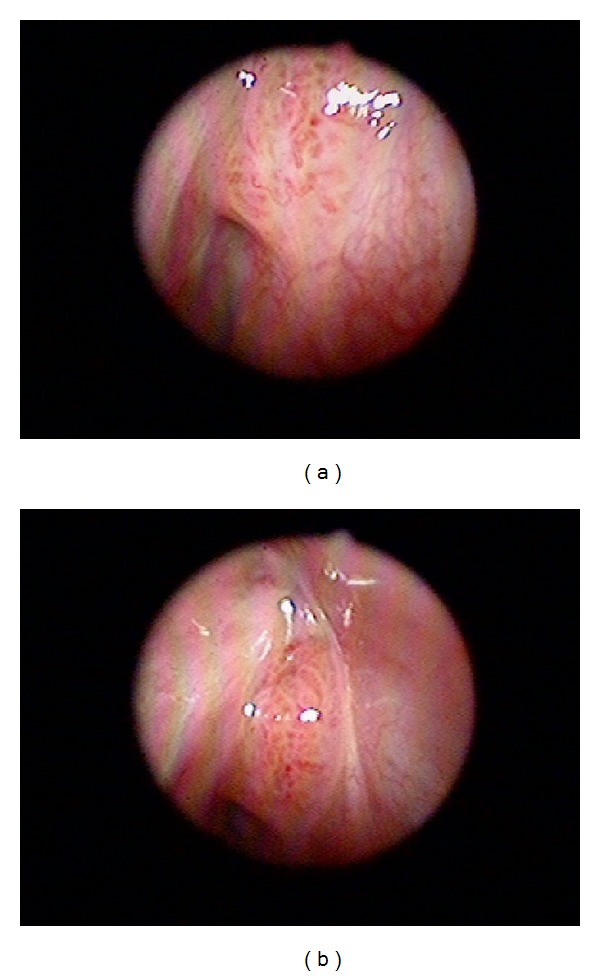
Sinoscopic view of sinonasal inverted papilloma with malignancy transformation.

**Figure 6:**
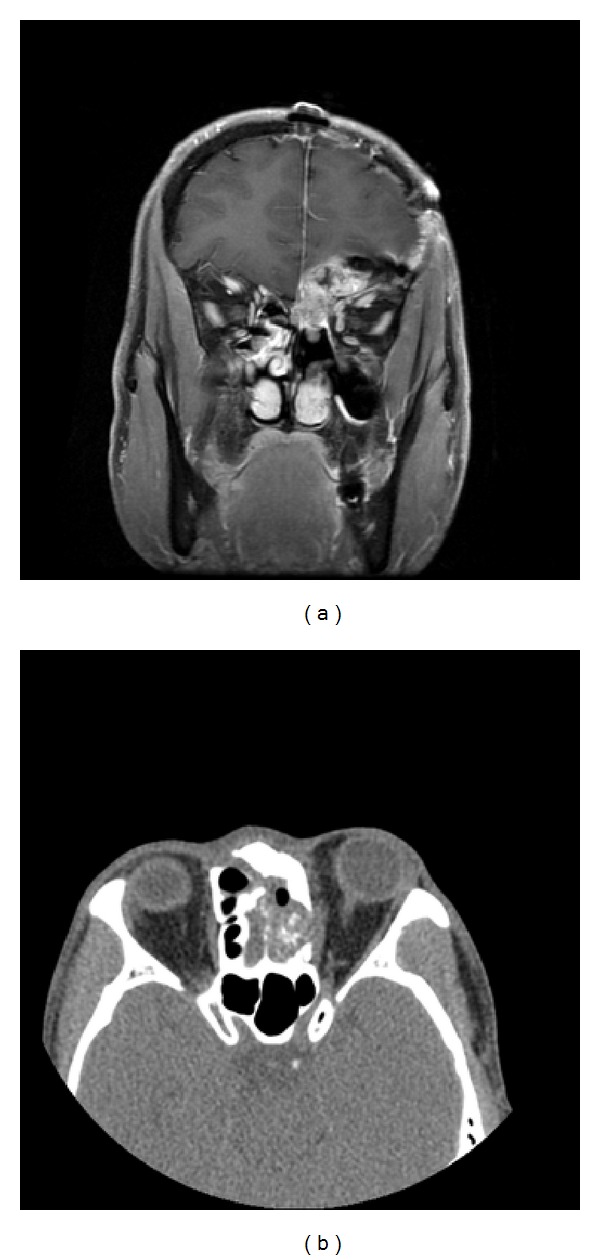
Sinus MRI (coronary view) and sinus CT scan (axial view) of sinonasal inverted papilloma with malignant transformation.

There were a total of 55 males and 15 females included in this study. Sixty of them are IPs and the other 10 are sinonasal IPs collected with malignant transformation. The age is 45.64 ± 14.45 years old ranging from 25 to 78 years old. The stains revealed significantly elevated levels of PLUNC, but decreased levels of Ki-67, p53, p21, and p27 in patients with multiple recurrence of IPs (Table 1). We also found the elevated PCNA, Ki-67, and p27 in the sinonasal IPs with squamous cell carcinoma transformation compared with sinonasal IPs alone with no synchronous malignancy (Table 2). The elevated PLUNC expression is correlated to multiple sinus surgery in the patients with sinonasal IPs. However, the PLUNC expression level is not correlated to the malignant transformation of IPs to SCC. We also showed the IHC expression of different levels for PCNA, Ki-67, p27, and PLUNC in Figures 1, 2, 3, and 4. The preoperative MRI or CT scan were all perform for all the patients as in Figures 5 and 6.

The elevated Ki-67 immunohistochemical staining is found in both sinonasal IPs with multiple recurrences and malignant transformation in our univariant and multivariant analysis by Pearson's chi-squared test and multiple logistic regression test as showed in Tables 1 and 2 and Figure 2(a). Therefore, high Ki-67 index could be considered an important factor for prognosis and malignant predicted markers. We could even combine the survey of the significantly increased Ki-67 and PCNA IHC expression level and the lower p27 level to predict the higher malignant transformation trend in patients with sinonasal IPs in our survey.

**Figure 7:**
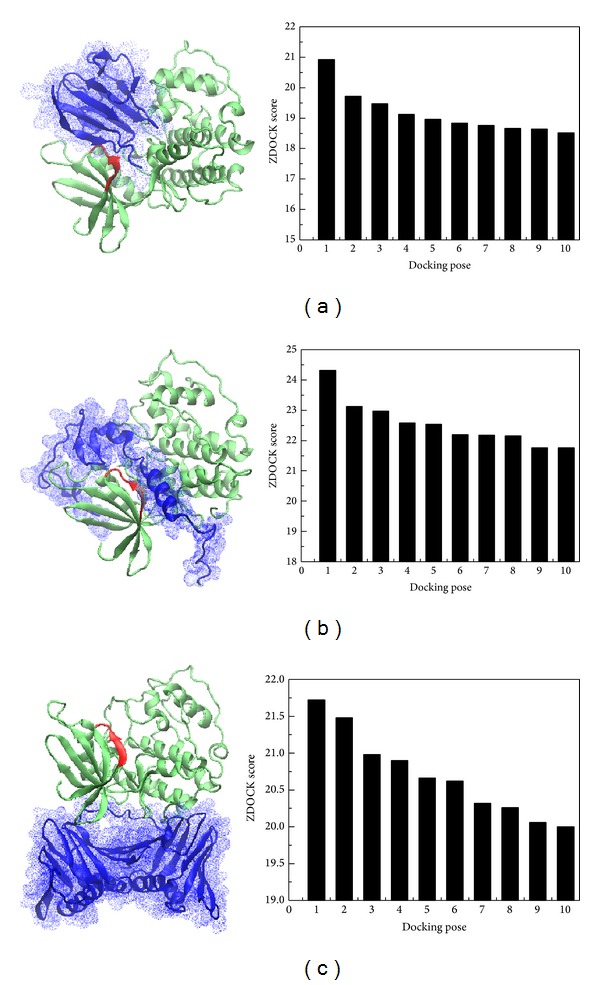
The best docking poses of CDK1 (green) with target protein (blue): (a) Ki-67, (b) p27, and (c) PCNA. The top ten protein-protein complexes with ZDOCK scores were generated by ZDOCK program. The highest ZDOCK score of Ki-67, p27, and PCNA are 20.92, 21.72, and 24.32, respectively. The key binding residues of Ki-67 and p27 are colored in red, and the key residues include Gly9, Ser10, Ile11, Leu12, Lys13, Lys14, and Val15.

**Figure 8:**
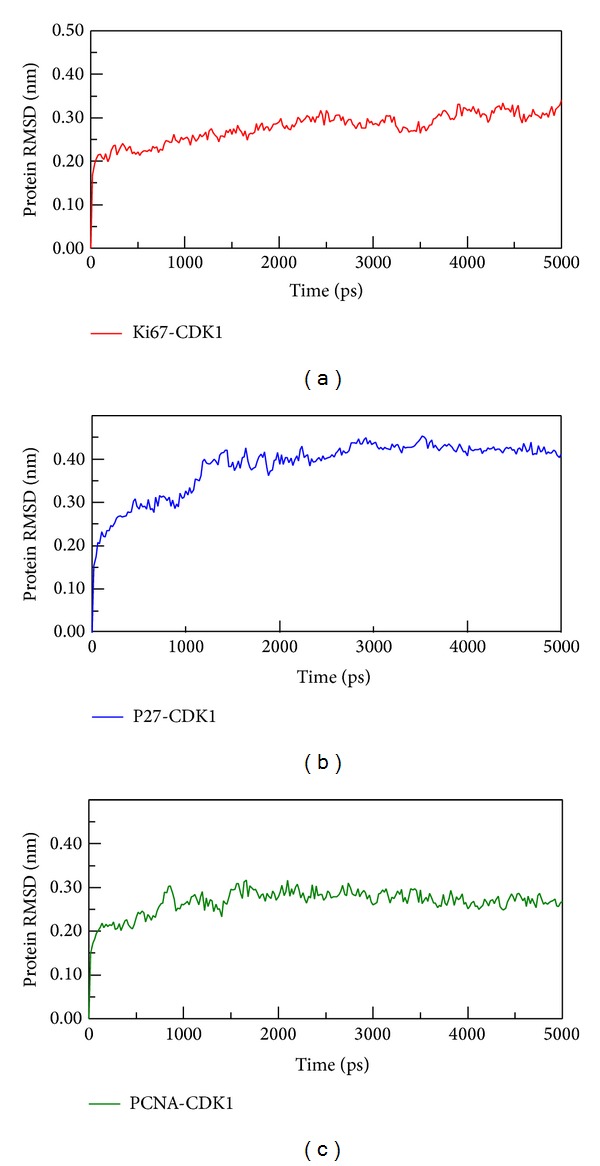
The RMSD analysis of all atoms of CDK1 complexes with (a) Ki-67, (b) p27, and (c) PCNA during 5000 ps simulation times. All complexes tend to stable fluctuation after simulation time of 1000 ps.

We consequently did some docking analyses with final molecular dynamic studies between what we found the prognosis factors of PCNA and CDK1, Ki-67 and CDK1, and p27 and CDK1 which all showed stable docking in Figure 7 and their docking score is also showed in by ZDOCK program. The ZDOCK generated the top 10 docking poses of CDK1 and Ki-67, CDK1-p27, and CDK1-PCNA. We chose the best docking score for analyzing the stability of protein-protein interaction. The Ki-67, p27, and PCNA to the CDK1 binding score are 20.92, 21.72, and 24.32, respectively by the ZDOCK program. The key residues are Gly9, Ser10, Ile11, Leu12, Lys13, Lys14, and Val15 which were the major binding domains for both Ki-67 and p27 to the CDK1 shown in red ribbon (Figure 7). We further did MD analysis for these key residues to see the interaction of these three proteins to the CDK1. After the MD simulation of CDK1 complex with Ki-67, p27, and PCNA, we performed the RMSD analysis for 5000 ps simulation times. All the three proteins (Ki-67, p27, and PCNA) are revealed to have stable fluctuation after 1000 ps simulation time (Figure 8).

**Figure 9:**
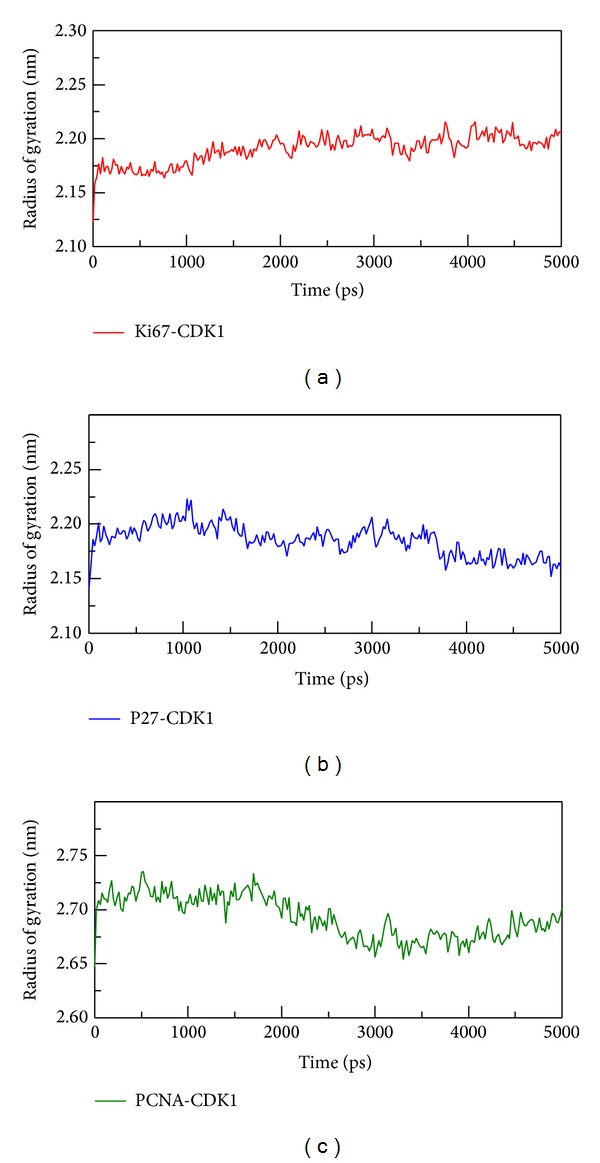
The radius of gyration of CDK1 complexes with (a) Ki-67, (b) p27, and (c) PCNA during 5000 ps simulation times. The low values of radius of gyration indicate the compacted complexes between two protein structures.

**Figure 10:**
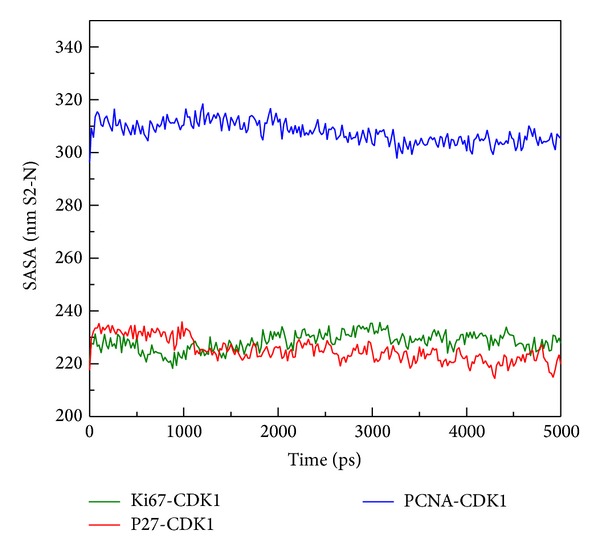
The SASA (area of solvent) analysis of all complexes conformation for hydrophobic definition during 5000 ps; the stable fluctuation indicated no distinct change between protein-protein interactions.

**Figure 11:**
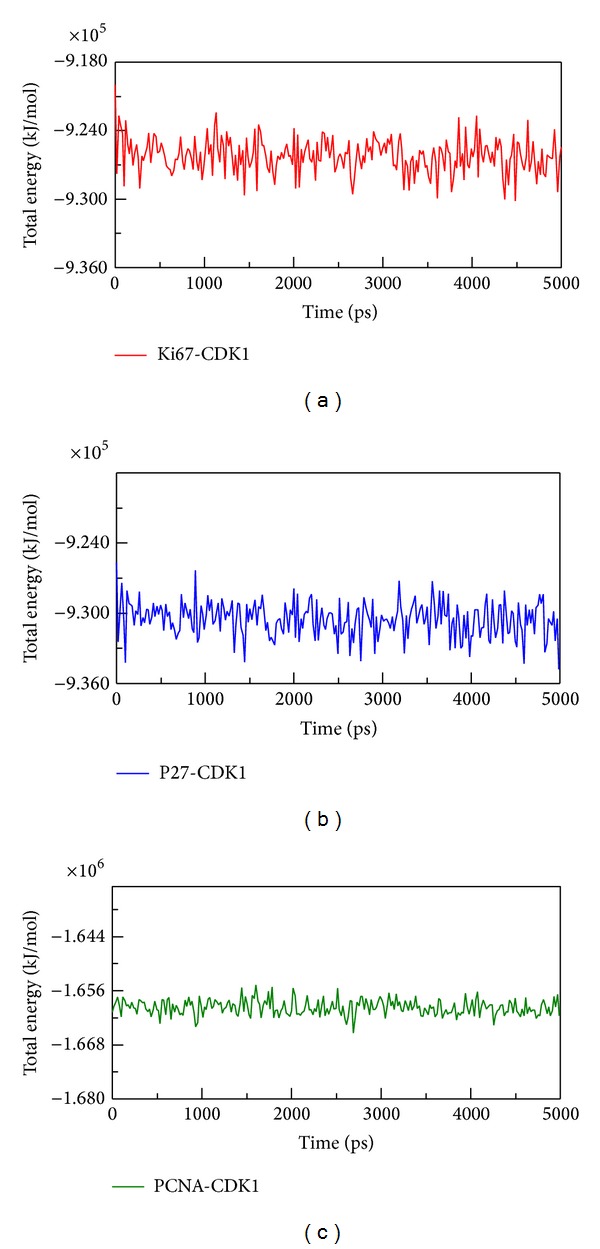
Total energy calculation of MD systems of (a) Ki-67, (b) p27 and (c) PCNA during 5000 ps simulation times, each of average fluctuations are −9.27 × 10^5^, −9.30 × 10^5^, and −1.66 × 10^5^, respectively.

**Figure 12:**
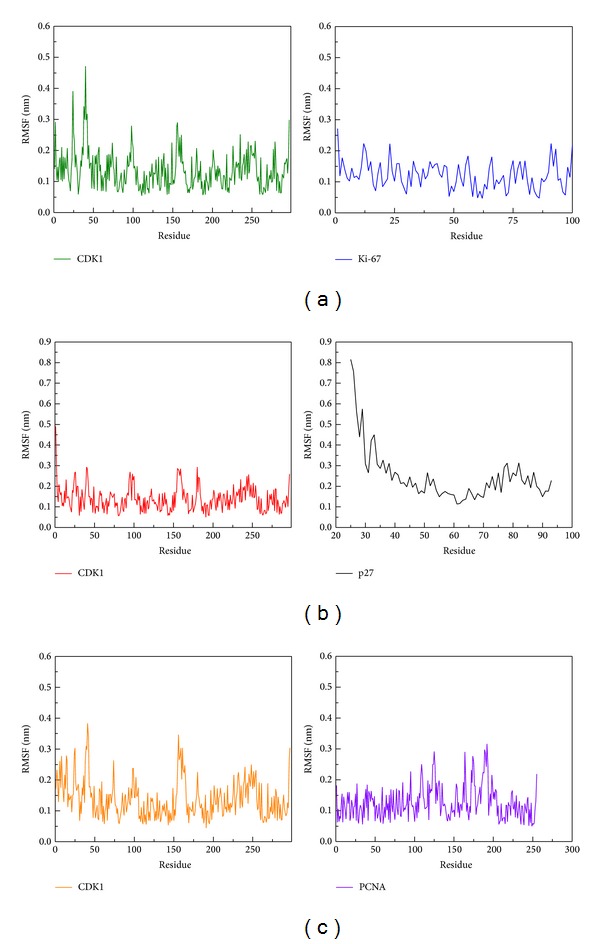
RMSF analysis of protein residues on (a) CDK1 and Ki-67, (b) CDK1 and p27, and (c) CDK1 and PCNA during simulation time of 5000 ps. The high value of RMSF fluctuation denotes strong variation of protein structure over all MD simulation.

In Figure 9, we calculated the proteins radius gyration for 5000 ps simulation time period. The three complexes tend to low values of radius of gyration and stable fluctuation over all MD simulation, which indicate the compacted complexes between each of the protein structure. The SASA (area of solvent) method was used for hydrophobic nature of the three complexes, these three became stable fluctuation and also showed stable hydrophobic change between each protein-protein interactions (Figure 10). Besides the total energy calculation of MD systems for each of the three complexes during 5000 ps simulation times, they all had stable energy variation in the range of −9.27 × 10^5^, −9.30 × 10^5^, and −1.66 × 10^6^, respectively (Figure 11). The result of energy analysis reveals that all simulation systems are stable during 5000 ps. In residues fluctuation analysis, we measure RMSF value of all residues for the three complexes (Figure 12). In RMSF validation of CDK1-Ki67 complex, there are significant fluctuations observed on residues from 38 to 43 of CDK1, which has great change during the MD simulation (Figure 12(a)). There was also significant variation observed in the residues from 25 to 40 on p27 protein structure during the MD simulation which denotes the great changes in this region. On the contrary, there is less RMSF variation in the residues from 100 to 200 on the PCNA protein structure during the MD simulation. Therefore, there was fewer RMSF change during MD simulation in PCNA than Ki-67 and p27.

**Figure 13:**
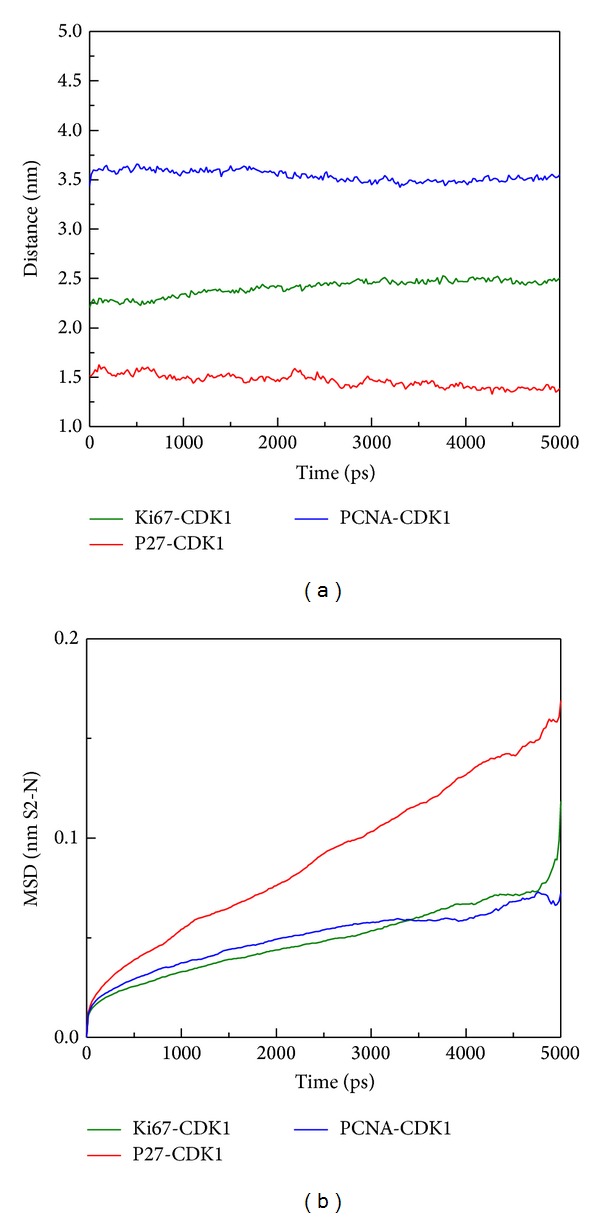
The migration analysis of complexes during simulation time of 5000 ps: (a) the distance between CDK1 and the docked proteins over all MD time and (b) the mean square displacement (MSD) analysis for all CDK1 complexes; the high value of MSD denotes the high distance of protein migration form initial position.

**Figure 14:**
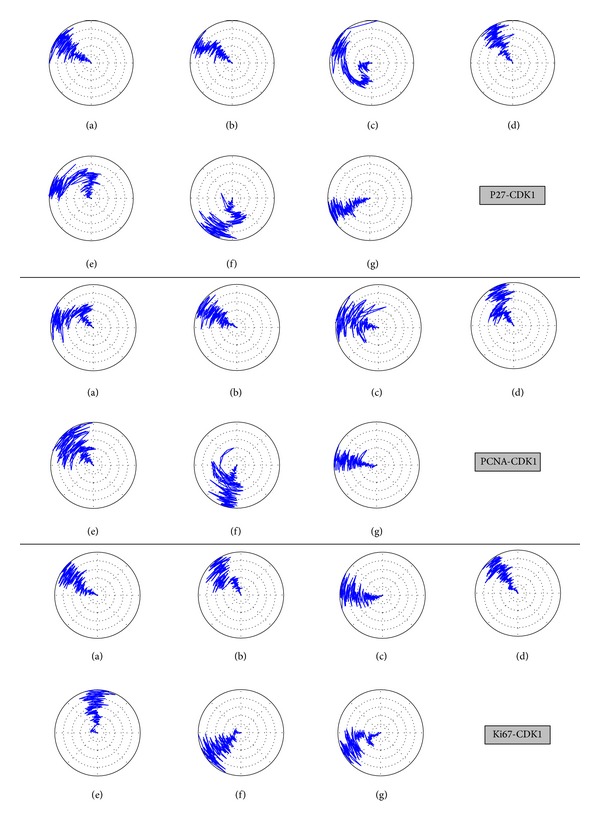
The dihedrals angle of key binding residues: (a) Gly9, (b) Ser10, (c) Ile11, (d) Leu12, (e) Lys13, (f) Lys14, and (g) Val15 on CDK1 protein structure during simulation time of 5000 ps. The dihedrals angles were calculated for CDK1 complexes with target binding proteins: Ki-67, p27, and PCNA over all MD simulation time.

**Figure 15:**
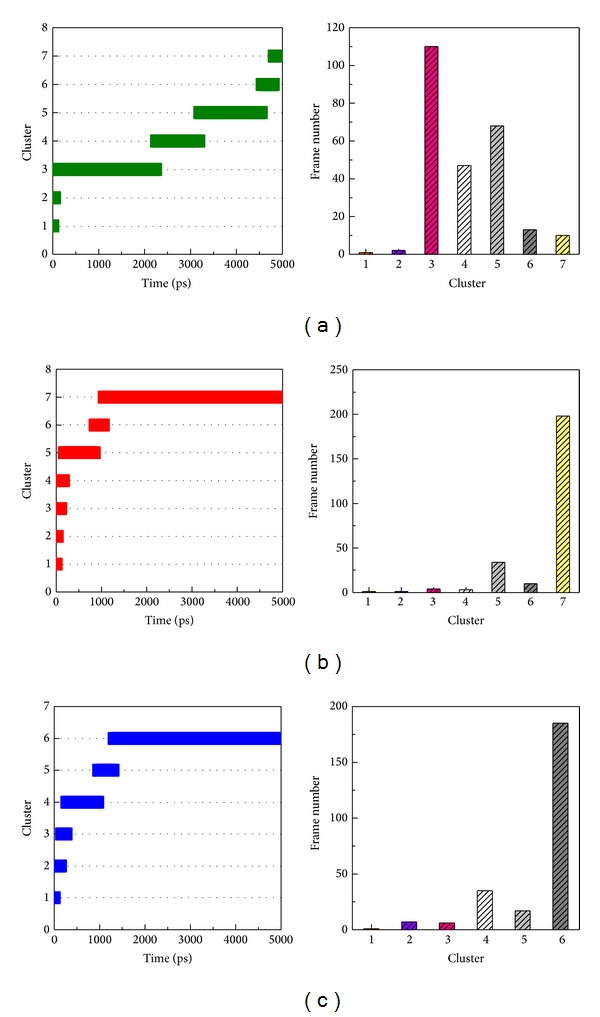
Cluster analyses of all CDK1 and the binding proteins: (a) Ki-67, (b) p27, and (c) PCNA during simulation time of 5000 ps; the represented structures were selected from the last clustering groups for all MD frames of CDK1 complexes. The represented structure of Ki-67, p27, and PCNA complex displaced at 4860 ps, 2740 ps, and 3880 ps, respectively.

**Figure 16:**
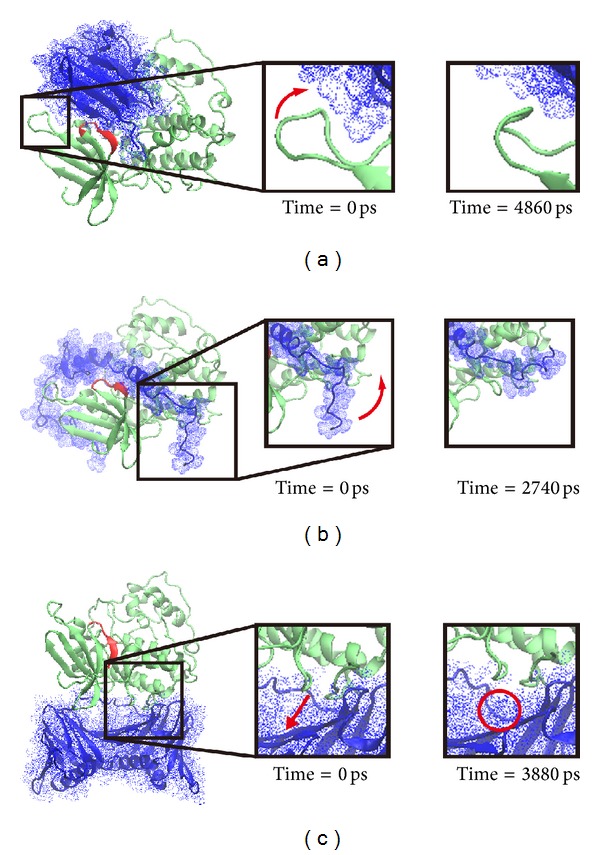
Comparison between the initial snapshot (0 ps) and represented conformation for all CDK1 complexes. (a) One of CDK1 (green) loop moved approach to Ki-67 (blue) at 4860 ps. (b) The protein structure of p27 (blue) bound to CDK1 (green) more tightly at 2740 ps. (c) One of PCNA (blue) loop moved away from CDK1 (green) at 3880 ps.

However, the migration analysis revealed that PCNA had great distance between CDK1 compared to Ki-67 and p27 during 5000 ps MD simulation survey. Although we found the largest distance between PCNA and CDK1 (Figure 13(a)), but their stable binding was shown in Figure 12 by RMSF analysis. In addition, the mean square displacement (MSD) of PCNA has smaller migration (Figure 13(b)). We further analyze the key residues of CDK1 for Ki-67 and p27 binding. The key binding residue includes Gly9, Ser10, Ile11, Leu12, Lys13, Lys14, and Val15 on Ki67-CDK1 protein structure dihedrals angle during simulation time of 5000 ps. All of the binding residues are stable during the whole MD simulation. However, there were only stable dihedrals angles in Gly9, Ser10, Leu12, and Val15 on p27-CDK1 protein complexes over all MD simulation time. Finally, we found that all the key binding residues including Gly9, Ser10, Ile11, Leu12, Lys13, Lys14, and Val15 on PCNA-CDK1 protein structure had stable binding dihedrals angle during the whole MD simulation; no greater changes were found during the process of PCNA binding to CDK1 (Figure 14). The Cluster analyses were used to select the representative structure among all MD frames. For snapshot comparison assay, the represented structure selected from the last clustering groups for all MD frames of CDK1 complexes of Ki67, P27, and PCNA displaced at 4860 ps, 2740 ps, and 3880 ps, respectively, during simulation time of 5000 ps (Figure 15). Snapshot comparison study for CDK1 and target proteins for Ki-67, p27, and PCNA are showed in Figure 16. We found that the residues of CDK1 from 38 to 43 on CDK1 are getting close to Ki-67 from 0 ps to 4860 ps (Figure 16(a)). The result is correlated to RMSF analysis due to the high fluctuations on residues from 38 to 43 of CDK1 binds for Ki-67. For CDK1-p27 snapshot analysis, p27 is getting close to CDK1 from 0 ps to 2740 ps. The findings are also correlated to RMSF analysis because of high variations on residues from 25 to 40 of p27 binds for CDK1 (Figure 16(b)). We could only find the small change of one PCNA (blue) loop move away from CDK1 (green) at 3880 ps at residues from 100 to 200 on PCNA and CDK1 interaction (Figure 16(c)). This result is also correlated to RMSF analysis for small fluctuations on residues from 100 to 200 of PCNA binds for CDK1.

**Figure 17:**
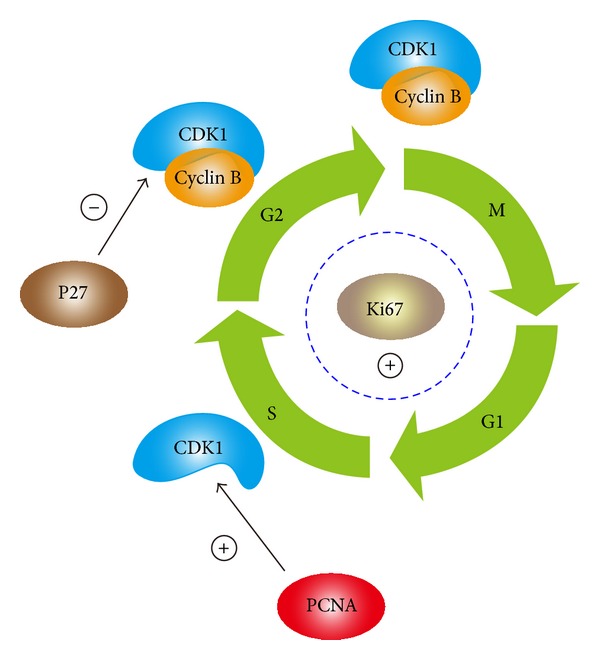
The molecular mechanism of Ki-67, p27, and PCNA in cell cycle.

To sum up the results in our study and literature reviews, the molecular mechanism of Ki-67, p27, and PCNA interacting with CDK1 for cells proliferation and malignant transformation in patients with sinonasal IP is showed in Figure 17.

## 4. Discussion

Although the inverted papillomas rarely occurred in the sinonasal tract, but the easy recurrent nature and malignant transformation often troubles patients and physicians. Life-long regular follow-up is needed for early detection of recurrence or malignant change and could lead to better disease control for IPs patients [9, 20].

The Ki-67 promoted the initiation of G1 phase from G0 in IPs cells, and it persisted expression for cell cycle in proliferation phase. Ki-67 is a protein, which affects cell cycle in the proliferation phase from G1-S-G2-M except the G0 phase [8, 11, 14]. In the literature reporting the p53, p63, p21, and p27 mutations induced sinonasal IP with SCC transformation, there were still debates [6, 11, 14]. Ki-67 is recently reported to the occurrence of sinonasal neoplasm [21]; the aggressive tumor behavior correlated to higher Ki-67 index and caused nasal epithelium to severe dysplasia and even squamous cell carcinoma [22]. The elevated Ki-67 could even be found in the IPs synchronously contained squamous cell carcinomas. Ki-67 will also affect p21, p27, and CDKs [8, 11, 12, 14]. However, we could not conclude whether the decreased p27 is caused by elevated expression of Ki-67 in sinonasal IPs tissues. Further study is warrant to elucidate the relationship between Ki-67 and P27.

The tumor suppression function of p53 is related to many cancers reported by Katori et al. and Gujrathi et al. [22, 23]. They suggested that testing for p53 may help to screen out papilloma lesions with a potential for dysplasia or carcinoma; however, we did not find it significant in multivariate analysis. This is because of the limited patient numbers in our study. However, not only p53 but also p63 is elevated expressed in IPs with malignant transformation [11].

Increased proliferative activity with elevated Ki-67 expression of tumor cells were reported as an important prognostic marker in many human tumors; it is also important in IPs recurrence and cancerization. Especially, Ki-67 suspect directly affects the cell cycle during proliferation phase and could interact with p53, p21, and p27 tumor suppressor genes and modulates cell cycle by affecting the checkpoint of G1 phase [8, 24, 25]. The Ki-67 recently found to have interaction between CDK1 in nature structure and molecular biology [26] and CDK1 partake the main role in cell proliferation and even malignant transformation [10]. We suspect that the Ki-67 not only initiated the IPs cells entrance into G1 phase of cell cycle but also caused malignant transformation in cell cores by affecting the CDK1.

The clinical roles of p21 and p27 to the head and neck SCC cancerization are still debates, but we found that the lower level of p27 also revealed in sinonasal IPs with malignant transformation in our study. Although there were few studies of p21 and p27 reporting the correlation to human IPs with cancerization, the debates still remained. Some were with Oncel et al. [11, 27] and some were against [25, 28] observed.

Besides Ki-67, we also found the PCNA, a predictor for IPs malignant transformation in the collaboration with CDK1. In our IPs with malignant transformation, both PCNA and Ki-67 were elevated by IHC stains.

As elevated PCNA expression showed in IP with malignant transformation from our survey, we suspected the PCNA to be important factor to induce cancerization for patients with sinonasal IP. Recently, it is also found the elevated PCNA in IP compared to the sinonasal polyps by Mumbuc et al. [3]. The PCNA could interact with CDK1 and promote the cell entry to the cell cycle to the consequent proliferation and cancerization.

Finally, we survey the PLUNC to the sinonasal IPs with cancerization since it is frequently reported to cause the nasopharyngeal cancer formation. In our prior study, the PLUNC expression was decreased in pseudomonas sinusitis in chronic disease status [29]. The PLUNC was also correlated to the chronic rhinosinusitis with multiple bacteria colonization [17]. And, besides, the PLUNC was also supposed to have anti-infection and antibiofilm function [30, 31]. Because it was supposed to have anticancerization of nasopharyngeal carcinoma [32], but we found no correlation of sinonasal IPs with SCC transformation. On the contrary, we could only find the elevated PLUNC expression in patients with sinonasal IPs with multiple recurrences and revision sinus surgery. The further mechanism and reasons for elevated PLUNC expression and multiple recurrences should be further surveyed in the future.

The Computer-Aided Drug design (CADD) can used to further investigate clinical or illness research, include illness research [33], risk factor studies [34], case reports, and molecular mechanism. In our docking and molecular dynamic results of PCNA, Ki-67, and p27 to CDK1, we found that all three of them had stable docking to the CDK1. And the p27 were supposed to have more stable interaction to the CDK1 to work as an inhibitor for sinonasal IPs with cancerization. The PCNA and Ki67 were considered as promoters and promote cell proliferation and cancerization in sinonasal IPs.

In conclusion, this is a first study showing that the Ki-67, PCNA, and p27 are all important in IPs recurrences and cancerization via CDK1. And we also are the first ones to find elevated PLUNC expression in multiple recurrence sinonasal IPs. However, further mechanism survey is still warranted in the future. The current computational studies are all compatible with our wet lab studies which make our study more trustable and could apply to the future treatment toward patients with such disease. Therefore, patients with sinonasal IP and expressed elevated Ki-67, PCNA, and decreased p27 should be considered to have higher possibilities of malignant transformation and should be followed more closely in clinical practice [35].

## Figures and Tables

**Table 1:** The correlation of IHC results with multiple recurrence in univariant by Pearson Xi square and Logistic multivariant regression analysis.

	>3 times surgery		Sum	*P *	pp
	0	1			
PCNA					
0	5	5	10	0.03	0.144
1	20	5	25		
2	15	10	25		
3	10	0	10		
Ki-67					
0	25	15	40	0.05	0.01
1	10	5	15		
2	10	0	10		
3	5	0	5		
P53					
0	10	15	25	0.0001	0.002
1	10	0	10		
2	10	0	10		
3	20	5	25		
P16					
0	20	5	25	0.04	1.000
1	10	10	20		
2	20	5	25		
3	0	0	0		
P21					
0	35	20	55	0.002	0.01
1	10	0	10		
2	5	0	5		
3	0	0	0		
P27					
0	10	10	20	0.02	0.04
1	15	0	15		
2	15	0	15		
3	20	0	20		
PLUNC					
0	10	0	0	0.02	0.002
1	5	0	0		
2	15	5	5		
3	20	5	15		

**Table 2:** The correlation of IHC results with malignant transformation in univariant by Pearson Xi square and Logistic multivariant regression analysis.

	Malignant transformation		Sum	*P *	pp
	0	1			
PCNA					
0	10	10	10	0.001	0.0001
1	25	25	25		
2	25	25	25		
3	0	10	10		
Ki-67					
0	40	0	40	0.001	0.0001
1	10	5	15		
2	10	0	10		
3	0	5	5		
P53					
0	25	0	25	0.001	0.187
1	5	5	10		
2	10	0	10		
3	20	5	25		
P16					
0	20	5	25	0.097	1.000
1	20	0	20		
2	20	5	25		
3	0	0	0		
P21					
0	50	5	55	0.002	0.214
1	5	5	10		
2	5	0	5		
3	0	0	0		
P27					
0	10	10	20	0.001	0.001
1	15	0	15		
2	15	0	15		
3	20	0	20		
PLUNC					
0	10	0	10	0.224	0.250
1	5	0	5		
2	15	5	20		
3	30	5	35		
